# *Streptococcus Suis* Serotype 2 Stimulates Neutrophil Extracellular Traps Formation via Activation of p38 MAPK and ERK1/2

**DOI:** 10.3389/fimmu.2018.02854

**Published:** 2018-12-07

**Authors:** Fang Ma, Xiaojing Chang, Guangyu Wang, Hong Zhou, Zhe Ma, Huixing Lin, Hongjie Fan

**Affiliations:** ^1^MOE Joint International Research Laboratory of Animal Health and Food Safety, College of Veterinary Medicine, Nanjing Agricultural University, Nanjing, China; ^2^National Center of Meat Quality and Safety Control, Nanjing Agriculture University, Nanjing, China; ^3^Jiangsu Co-Innovation Center for Prevention and Control of Important Animal Infectious Diseases and Zoonoses, Yangzhou University, Yangzhou, China

**Keywords:** *Streptococcus suis* serotype 2, neutrophil extracellular traps, reactive oxygen species, p38 MAPK, ERK1/2, TLR4 signaling

## Abstract

*Streptococcus suis* serotype 2 is a major pathogen of swine streptococcicosis, which result in serious economic loss worldwide. SS2 is an important zoonosis causing meningitis and even death in humans. Neutrophil extracellular traps (NETs) constitute a significant bactericidal strategy of innate immune. The battle between SS2 and NETs may account for the pathogenicity of SS2. However, the molecular mechanism underlying release of SS2-induced NETs remains unclear. In this study, SS2 was found to induce NETs within 2–4 h, and was dependent on reactive oxygen species (ROS) from NADPH oxidase. Moreover, SS2 could activate neutrophil p38 MAPK and ERK1/2. Blockage of p38 MAPK or ERK1/2 activation decreased SS2-induced NETs formation by 65 and 85%, respectively. In addition, NADPH oxidase derived ROS inhibition negatively affected phosphorylation of p38 MAPK and ERK1/2 in SS2 induced neutrophils. Both TLR2 and TLR4 were significantly up-regulated by SS2 infection in blood cells *in vivo* and neutrophils *in vitro*, which indicates these two receptors are involved in SS2 recognition. Blocking TLR4 signaling could further inhibit the activation of ERK1/2, but not p38 MAPK; however, TLR4 signaling inhibition reduced NETs formation induced by SS2. In conclusion, SS2 could be recognized by TLR2 and/or TLR4, initiating NETs formation signaling pathways in a NADPH oxidase derived ROS dependent manner. ROS will activate p38 MAPK and ERK1/2, which ultimately induces NETs formation.

## Introduction

*Streptococcus suis* serotype 2 (SS2) is a swine pathogen responsible for various diseases including meningitis, septicemia and even acute death; it is an important zoonotic pathogen causing serious invasive infections in humans worldwide. In addition, SS2 is the main pathogen of human meningitis in some regions and countries in Asia (1). People can contract SS2 via contact with diseased pigs or consumption of contaminated pork, which indicates that SS2 has evolved a variety of significant strategies to evade the host's innate immune system (2, 3).

Neutrophils are the most abundant immune cells among white blood cells, and play a vital role in defense against invasive pathogens and Neutrophil extracellular traps (NETs) are regarded as a significant bactericidal mechanism of innate immune system (4–6). NETs are reticular fiber structures consisting of nuclear constituents and abundant bactericidal proteins, which will efficiently entrap and kill bacteria (7). A variety of pathogens are reported to elicit NETs formation, and include *Staphylococcus aureus* (*S. aureus*) and Group A streptococcus (8, 9). In addition, bacteria, viruses, fungi, and such cytokines such as IL-8 can activate neutrophils and stimulate NETs release (4, 10, 11).

It is reported that various stimuli induce NETs formation through different mechanisms (12). There are three models of NETs formation reported to date and the most classic model is suicidal NETosis, which is a type of cell death caused by NETs release (13). In the classic model, NETs formation is dependent on the generation of ROS through the activation of NADPH oxidase complex and this process is within 2–4 h (14). Many stimuli, such as PMA, Group B Streptococcus, *Candida albicans, Leishmania*, and *Mycobacerium tuberculosis*, have been reported to induce NETs formation; this is dependent on ROS produced from NADPH oxidase, which acts as a second manager to promote chromosome decondensation (15–20). However, ROS is not necessary in some processes of NETs formation. Another model proposes that NETs occurs independently of ROS and without loss of nuclear or plasma membrane (21). For example, lipopolysaccharide (LPS) stimulated NETs formation involving TLR4 on platelets was independent on ROS release (22). *S. aureus* could induce a rapid NETs release within 10 min, which was an insufficient time to detect ROS (23). In addition, a third model describes NETs release as occurring within 15 min, and the NET backbone is derived of mitochondrial DNA instead of nuclear DNA (24).

The mechanism of NETs induction are not fully understood. The Raf/ERK pathway was first reported involved in NETs formation induced by phorbol 12-myristate 13-acetate (PMA) (25). Extracellular signal related kinase 1/2 (ERK1/2), p38 MAPK, and stress-activated protein kinase (SAPK/JNK) are three major characterized MAPK families to date (26). MAP kinases are central to signal transduction pathways, and there are reports that MAP kinases are involved downstream of NETs formation induced by many stimuli (27–29). Whether MAP kinases play roles in SS2-induced NETs formation needs to be explored.

The interaction between NETs and SS2 is complicated and unclear, and Zhao et.al found that NETs played an important role in clearance of SS2 *in vivo* and *in vitro* (30, 31). In addition, NETs have also been detected in cerebrospinal fluid of SS2 infected piglets (32). SS2 induced NETs formation occurs at an early time point (120 min), which indicated that it likely belongs to the classic model (31); this model was further explored in this study. A previous study showed that SS2 could form a biofilm to protect bacteria from phagocytosis, however, SS2 could still be entrapped and killed by NETs (33). Meanwhile, SS2 was shown to inhibit NETs release with an extracellular biofilm matrix; however, the molecular mechanism of this form of NETs inhibition remains unclear (33). In addition, the signaling pathways downstream of SS2-induced NETs formation are still unclear to date. Therefore, understanding the molecular mechanism of SS2-induced NETs formation will provide a theoretical basis to explain some challenges of NETs inhibition induced by SS2 and other pathogens, which will of benefit for the further study of SS2 pathogenicity.

The checks and balances between host and pathogen determine the development of disease caused by SS2. This study aims to explore the molecular mechanism of NETs release induced by SS2, which will provide knowledge to further understand the strategies of SS2 pathogenicity from the perspective of NETs induction. Here, the role of neutrophil ROS, p38 MAPK, ERK1/2, JNK/SAPK, and the major neutrophil cell surface TLRs associated with bacterial recognition in the process of NETs formation induced by SS2 are explored and confirmed.

## Materials and Methods

### Ethics Statement

This study was carried out in accordance to animal welfare standards and were approved by the Ethical committee for Animal Experiments of Nanjing Agricultural University, China. All animal experiments accorded with the guidelines of the Animal Welfare Council of China.

### Bacterial Strains and Experimental Animals

The wild-type SS2 strain ZY05719 was isolated from Jiangsu Province, and was grown in Todd-Hewitt broth (THB) medium (Difco, BD, Franklin, NJ, USA) at 37°C on a gentle rocking shaker.

Four-week-old female Institute of Cancer Research (ICR) specific-pathogen-free mice were purchased from the Comparative Medicine of Yangzhou University. Six-week old female wild-type (WT) B10 mice and TLR4 knockout (KO) mice on a B10 background were purchased from Model Animal Research Center of Nanjing University. All experimental protocols were conducted according to animal welfare standards, and were approved by the Ethical Committee for Animal Experiments of Nanjing Agriculture University, China.

### Neutrophils Isolation

Neutrophils were isolated from 4 week old ICR mouse bone marrow as previously described (33). Briefly, tibias and femurs were collected from euthanized mice, and then bone marrow was flushed with sterile PBS into a 15 mL tube (BD Falcon). The cells were washed and resuspended in 3 mL of PBS. In a new 15 mL tube, a Percoll gradient was prepared by carefully overlaying 3 mL of 80% Percoll, 3 mL of 65% Percoll, and 3 mL of 55% Percoll. The cell suspension was then overlaid on top of the Percoll and centrifuged at 1000 × *g* for 30 min. Thereafter, the cells were collected at the 80/65% gradient interface, and then washed and suspended in RPMI1640 medium. Purity of neutrophils were determined by flow cytometry with FITC-Ly6G and PE-CD11b labeled.

### NETs Quantification

The NETs quantification assay was performed as previously described with some modifications (34). Neutrophils were incubated on 96 well-plates and were infected with SS2 ZY05719 at a MOI of 10. NET DNA backbone was quantified with a Quant-iT Picogreen ds DNA assay kit (Invitrogen). After incubation of bacteria and neutrophils, 0.5 U/ml of micrococcal nuclease (MNase) was added to each well to release NETs bound DNA. After incubation for 10 min, 10 mM EDTA was added to terminate the reaction and the plate was centrifuged at 400 × *g* for 10 min. An aliquot (100 μL) of supernatant was thoroughly mixed with 100 mL of working solution. After 5 min incubation, the fluorescence was read with a multifunctional microplate reader (Tecan Infinite Pro) at 480 nm excitation and 520 nm emission.

### NETs Visualization

NETs visualization was performed as previously described (35). The cells were cultured on poly-L-lysine-coated cover slides. SS2 ZY05719 was cultured to the mid-exponential phase and collected in PBS. An aliquot (100 μL) of previously isolated bacteria-infected neutrophils (MOI of 10) were centrifuged at 400 × *g* for 10 min. Neutrophils incubated with 200 nM PMA served as positive control. After 3 h incubation, the cover slides were fixed with 4% paraformaldehyde for 10 min, permeabilized with 0.1% Triton X-100 and then blocked with 10% goat serum at 4°C overnight. The sample were stained with primary anti-Neutrophil Elastase antibody (1:50 diluted, Abcam, Cambridge, UK) at 4°C overnight, followed by incubation with goat anti-rabbit Alexa 568 antibody (1:100 dilution, Jackson ImmunoResearch, West Grove, PA, USA) for 1 h at room temperature in the dark. The DNA was stained with 4′,6-diamidino-2-phenylindole (DAPI, Thermo Fisher). The images were recorded using a confocal microscope (Zeiss, Germany).

### Reactive Oxygen Species (ROS) Assay

Production of ROS from neutrophils induced by SS2 ZY05719 was determined by oxidation of 2,7-dichloroflurescin diacetate (DCFH-DA) to fluorescent dichlorofluorescin (DCF) (36). Neutrophils were infected with SS2 ZY05719 at a MOI of 10 for 2 h, and then the cells were loaded with 100 μM DCFH-DA for 30 min. Cells were washed 3 times to remove DCFH-DA. The fluorescence was measured and recorded in a multifunctional microplate reader at excitation and emission wavelengths of 488 and 520 nm, respectively. For detection by fluorescent microscope detection, neutrophils with DCFH-DA were cultured on PLL-treated cover slides, infected with bacteria for 3 h, and then visualized with a fluorescent microscope (Zeiss Germany).

### Inhibition Assay

The inhibition assay was performed as previously described (37). Briefly, neutrophils were separately treated with 20 μM of p38 MAPK inhibitor SB203580 (MCE, Monmouth Junction, NJ, USA), 10 μM of MEK inhibitor U0126 (MCE), 10 μM of NADPH oxidase inhibitor diphenyleneiodonium chloride (DPI, Sigma Aldrich), and 10 μM TLR4 inhibitor TAK-242 (Sigma Aldrich, St. Louis, MO, USA) for 30 min at 37°C. SS2 ZY05719 (MOI = 10) or PMA (200 nM) was added to the pretreated cells for 3 h. Subsequently, the samples were collected for laser confocal microscopy, NETs quantification and western blotting detection.

### Western Blotting

Neutrophils with or without inhibitor pretreatment were stimulated with SS2 ZY05719 (MOI = 10) or PMA (200 nM) for 2 h, and then cells were lysed for 10 min on ice with RIPA plus 1% protease inhibitor cocktail (ApexBio, Houston, USA) and 10% PhosStop (Roche Life Science, Basel, Switzerland). The lysates were mixed with loading buffer, and boiled at 100°C for 10 min. Samples were resolved on 12% SDS-PAGE gels, and transferred to PVDF membranes. The membranes were blocked with 5% skimmed milk/TBST at 4°C overnight, and washed three times with PBST. Thereafter, the membranes were incubated with Phospho-p38 MAPK (Thr180/Thr182) (D3F9) XP ® Rabbit mAb, p38 MAPK (D13E1) XP® Rabbit mAb, Phospho-p44/42 MAPK (Erk1/2) (Thr202/Tyr204) (D13.14.4E)XP ® Rabbit mAb, p44/42 MAPK (Erk1/2)(137F5) Rabbit mAb, Phospho-SAPK/JNK (Thr183/Tyr185) (81E11) Rabbit mAb (1:1000, Cell Signaling Technology, Danvers, MA, USA), or anti-GAPDH Rabbit pAb (1:5000, CMCTAG, Milwaukee, WI, USA) at 4°C overnight. After washing with PBST, the membranes were incubated with HRP-Goat Anti-Rabbit IgG (H+L) (1:5000, CMCTAG) antibody for 1 h at room temperature. The reaction complexes were detected using an enhanced chemiluminescence ECL kit (CMCTAG). To further explore the role of TLR4 in the activation of p38 MAPK and ERK1/2, neutrophils from TLR4 KO mice and WT mice were infected with SS2 ZY05719 for 2 h and then the samples were determined with western blotting as above.

### qRT-PCR

To determine the transcriptional levels of TLRs *in vivo*, mice were infected with SS2 ZY05719 via intravenous caudal vein injection, and 200 μL of blood (heparin anticoagulated) was collected at 1, 2, 3, 4, 5, and 6 h post infection. Then, 1 mL of Trizol was immediately added to the blood. To determine the transcriptional level of TLRs *in vitro*, neutrophils were infected with SS2 ZY05719 at a MOI of 10 for 2 and 3 h and 1 mL of Trizol was added to each sample.

The Trizol mixture was vortexed for 15 s to lyse the cells and then 200 μL of chloroform was added. The mixture was centrifuged at 12,000 × *g* for 15 min. Thereafter, 200 μL of the upper phase was collected, and 500 μL of isopropyl alcohol was added and incubated at room temperature for 30 min to precipitate the RNA. The suspension was centrifuged at 12,000 × *g* for 15 min to collect the RNA, which was washed twice with 75% ethanol. The remaining genome DNA was digested with DNase I (Fermentas, Thermo Fisher Scientific, Wilmington, Delaware USA). The concentration and quality of RNA were determined with Thermo NanoDrop2000 (Thermo Fisher Scientific) and agarose gel electrophoresis.

Complementary DNA was synthesized from 2 μg of total RNA using the PrimeScript^TM^ II 1st Strand cDNA Synthesis Kit (TaKaRa). mRNA levels of TLR2, TLR4, and TLR6 were measured and analyzed with TB Green^TM^ *Premix Ex Taq*^*TM*^ II (Tli RNaseH Plus), ROX plus (TaKaRa) and Applied Biosystems StepOne^TM^ Real-Time PCR System. The primer combinations are listed in Table 1. The GADPH gene was used as an internal reference, and the relative changes of gene expression were normalized with GADPH gene using the 2^−^ΔΔCt method (38).

**Table 1:** Primers in this study.

**Primer**	**Sequence (5^**′**^-3^**′**^)**
Fl-TLR2F	ACTGTGTTCGTGCTTTCTGAG
Fl-TLR2R	ATGGCTTTCCTCTCAATGGG
Fl-TLR4F	GAGGACTGGGTGAGAAATGAG
Fl-TLR4R	GTAGTGAAGGCAGAGGTGAAAG
Fl-TLR6F	GTCAAGAACATAGGCTGGGTAG
Fl-TLR6R	GCAGAACAGTATCACAGGACAG
GADPHF	CCACTCACGGCAAATTCAAC
GADPHR	CTCCACGACATACTCAGCAC

### Statistical Analysis

All experiments were repeated at least 3 times. The Prism 5 software package (GraphPad Software, La Jolla, CA, USA) and SPSS were used to perform statistical analyses. *P*-values of < 0.05 were considered statistically significant.

## Results

### NETs Formation Is Induced by SS2 at 120 min Post-infection

**Figure 1:**
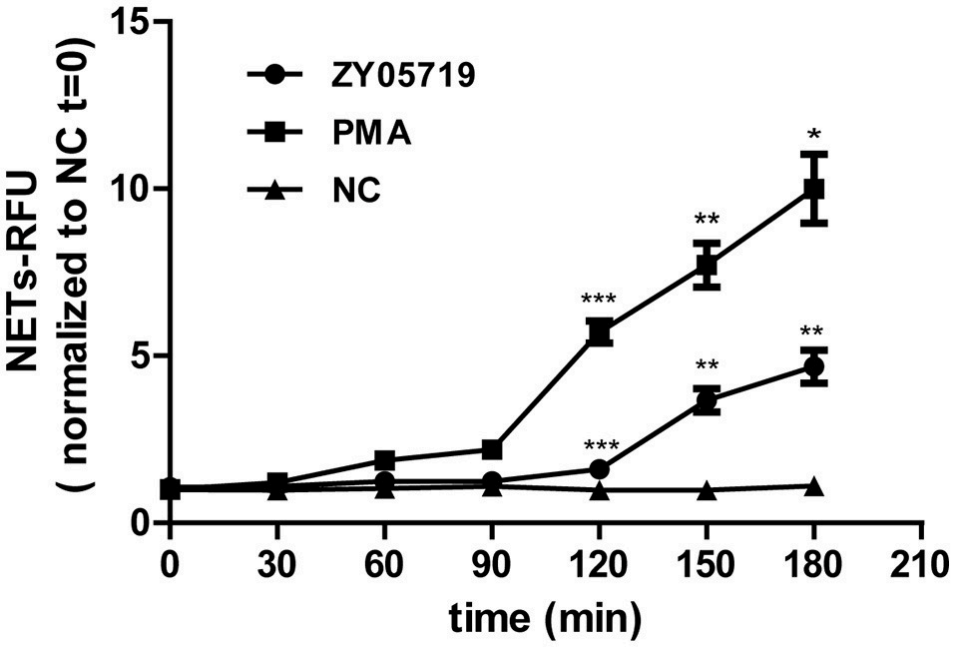
NETs formation induced by SS2. Neutrophils extracellular DNA was assayed with Picogreen at 30, 60, 90, 120, 150, and 180 min post-infection with SS2 ZY05719; PMA was used for neutrophils induction as the positive control. NETs formation was measured as the concentration of extracellular DNA, which was represented as relative fluorescence units (RFU) and normalized to non-activated neutrophils (NC, *t* = 0). The statistical analyses were between group ZY05719 and NC or group PMA and NC at a given time point. Results are depicted as the mean ±SEM (*n* = 3) of three independent experiments. ^*^p < 0.05; ^**^p < 0.01; ^***^p < 0.001; ns, no differences between groups.

SS2 was reported to stimulate NETs release *in vivo* and *in vitro* (31, 33). In this study, neutrophils were infected with SS2 ZY05719, and extracellular DNA which could represent NETs formation was detected at 30, 60, 120, and 180 min post-infection. This result showed that SS2-induced NETs could be observed only at 180 min post infection (Figure 1), demonstrating that SS2-induced NETs release is not a quick process (within 15 min); this indicated that SS2-induced NETs formation likely belongs to the within 2–4 h classic model.

### Roles of ROS in SS2-Induced NETs Formation

**Figure 2:**
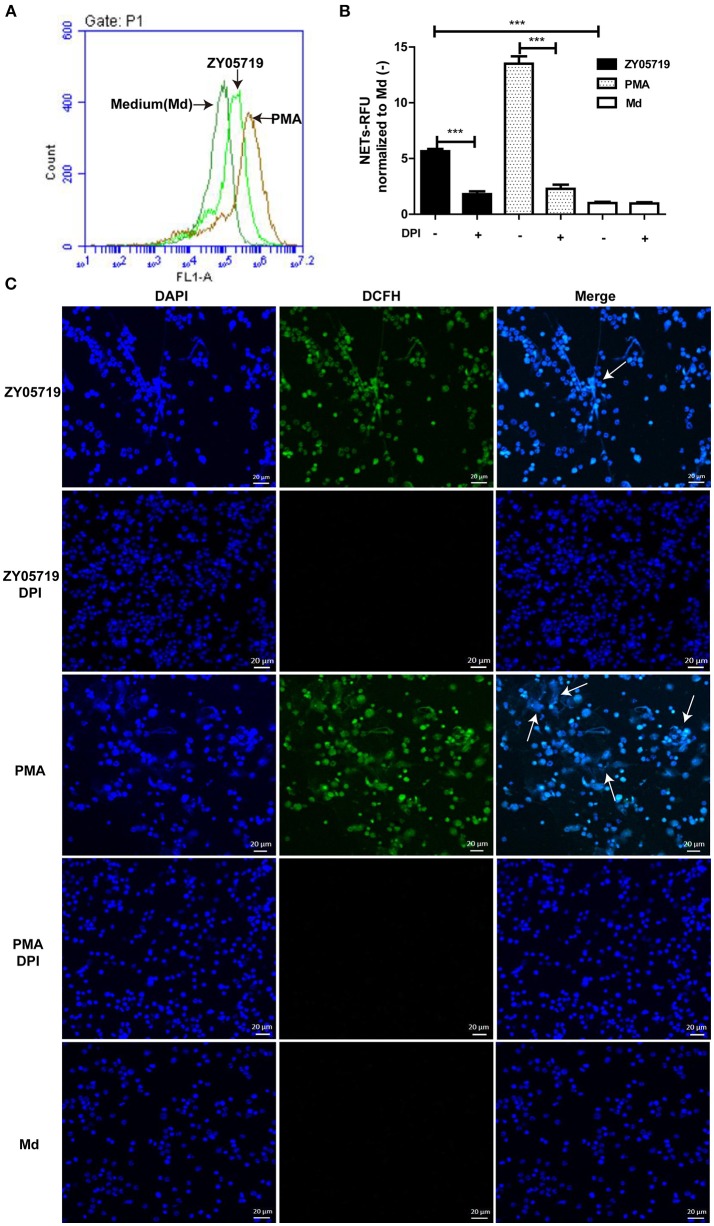
Roles of NADPH oxidase derived ROS in SS2-induced NETs release **(A)** Neutrophils were incubated with PMA or SS2 ZY05719 for 2 h, and then loaded with DCFH-DA. ROS generation will catalyze DCFH-DA to green fluorescence DCFH, which was detected by flow cytometry; the X-axis represents fluorescence intensity and the Y-axis represents cell counts. **(B)** After pretreatment with inhibitors of NADPH oxidase (DPI), neutrophils were incubated with PMA and SS2 ZY05719 for 3 h. Results were normalized to neutrophils without pretreatment and activation, but were loaded with DCFH-DA and are depicted as the mean ±SEM (*n* = 5) of three independent experiments; ^***^p < 0.001; **(C)** After pretreatment with DPI, neutrophils were infected with SS2 ZY05719 and PMA for 3 h. Untreated and uninfected neutrophils were loaded with DCFH-DA as a negative control. ROS generation was labeled with DCFH (green) and DNA was stained with DAPI (blue). The arrows indicating NETs structure. The results shown are representative of three independent experiments.

To further determine whether SS2-induced NETs formation belongs to the classic model, ROS generation was assayed. Flow cytometry results showed that ROS generation was increased in SS2 ZY05719 induced neutrophils compared with the untreated cells (Figure 2A). When the NADPH oxidase ROS source was inhibited by DPI, both SS2- and PMA-induced NETs were inhibited significantly (Figure 2B). ROS and NETs formation was visualized with laser confocal microscopy; ROS and NET DNA backbone were observed both in SS2- and PMA-stimulated neutrophils (Figure 2C). However, the formation of ROS and NETs were suppressed with addition of DPI (Figure 2C). These results indicated that SS2-induced NETs release required NADPH-oxidase produced ROS.

### Effects of p38 MAPK and ERK1/2 Activation on SS2-Induced NETs Release

**Figure 3:**
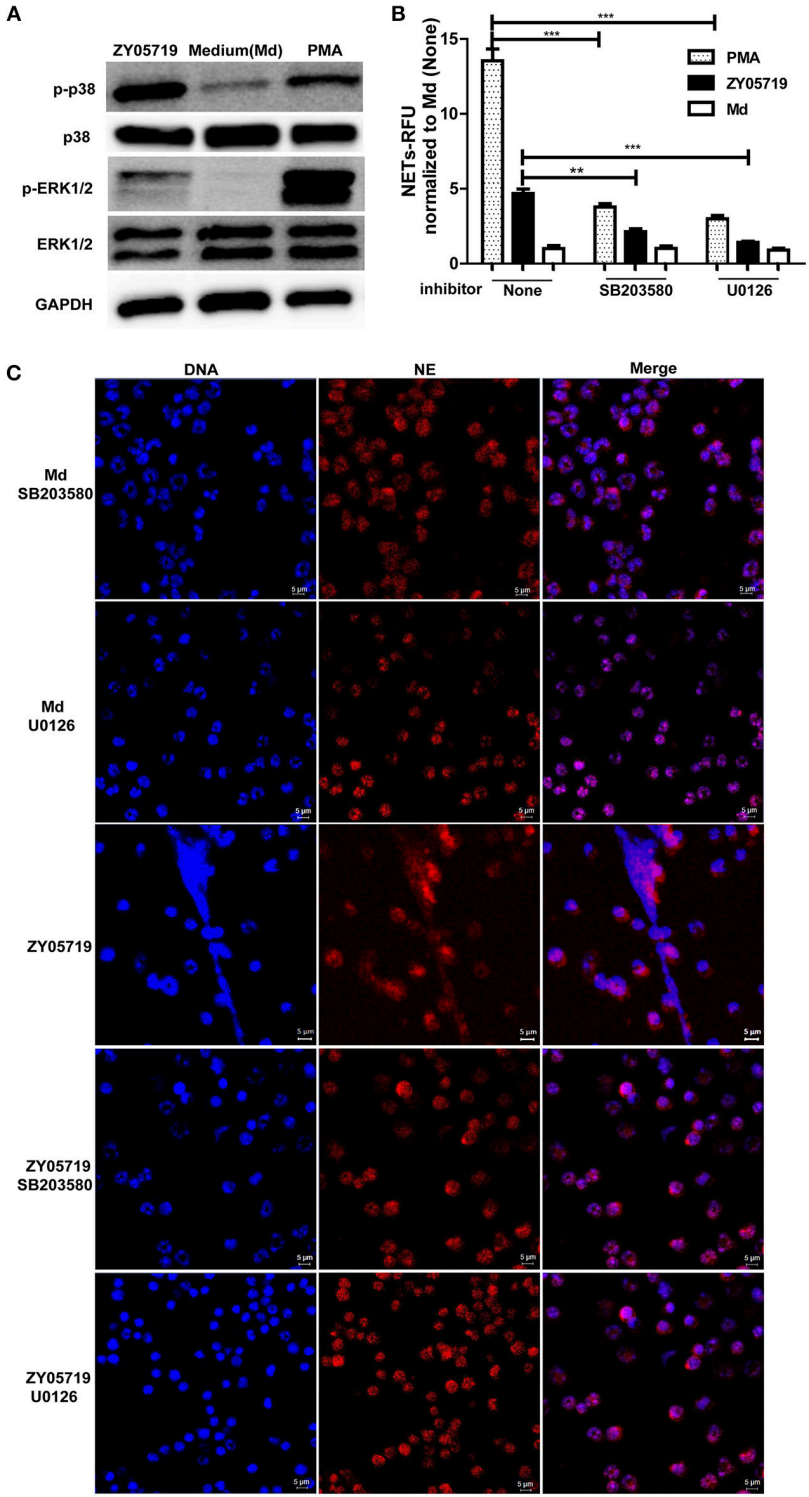
Role of the activation of p38 MAPK and ERK1/2 in SS2 and PMA induced NETs formation. **(A)** Neutrophils were infected with SS2 ZY05719 or treated with PMA for 2 h; p38 MAPK and ERK1/2 phosphorylation were determined with western blotting using specific antibody against phospho-p38 MAPK (p-p38), p38 MAPK (p38), phospho-ERK1/2 (p-ERK1/2), and ERK1/2. GAPDH was used as an internal control. **(B)** Neutrophils were pretreated with inhibitor of p38 MAPK (SB203580) and ERK1/2 (U0126), and then cells were incubated with SS2 ZY05719 or PMA for 3 h. Results were normalized to neutrophils without pretreatment and activation, and are depicted as the mean ±SEM (*n* = 3) of three independent experiments; ^**^p < 0.01; ^***^p < 0.001. **(C)** Neutrophils were pretreated with inhibitor of p38 MAPK (SB203580) and ERK1/2 (U0126), and the cells were then incubated with either SS2 ZY05719 or PMA for 3 h. Immunofluorescence was performed using anti-neutrophil elastase (NE) antibody followed by goat anti-rabbit Alexa 568 antibody (red). DNA was stained with DAPI (blue). The results shown are representative of three independent experiments.

To detect whether MAPKs-p38, ERK and JNK were involved in SS2-induced NETs formation, neutrophils were infected with SS2 ZY05719 for 2 h, and phosphorylation of p38 MAPK, ERK1/2, and JNK/SPAK were then determined by western blot. When neutrophils were infected with SS2 ZY05719, p38 MAPK, and ERK1/2 were both phosphorylated, indicating that p38 MAPK and ERK1/2 were activated in the process of SS2 infection (Figure 3A). However, activation of JNK/SPAK were not observed (data not shown). To further determine the function of neutrophil p38 MAPK and ERK1/2 activation on NETs formation, neutrophils were separately pretreated with inhibitors SB203580 and U0126 to suppress the activation of p38 MAPK and ERK1/2. Neutrophils were then infected with SS2, and NETs formation was determined with a fluorescence microplate reader and fluorescence microscopy. The results demonstrated that inhibiting the activation of p38 MAPK and ERK1/2 would suppress SS2 induced NETs formation to some degree (Figures 3B,C). In summary, SS2 infection could activate p38 MAPK and ERK1/2, and both p38 MAPK and ERK1/2 phosphorylation play important role in SS2-induced NETs formation.

### Transcriptional Levels of TLR2 and TLR4 During SS2 Infection

**Figure 4:**
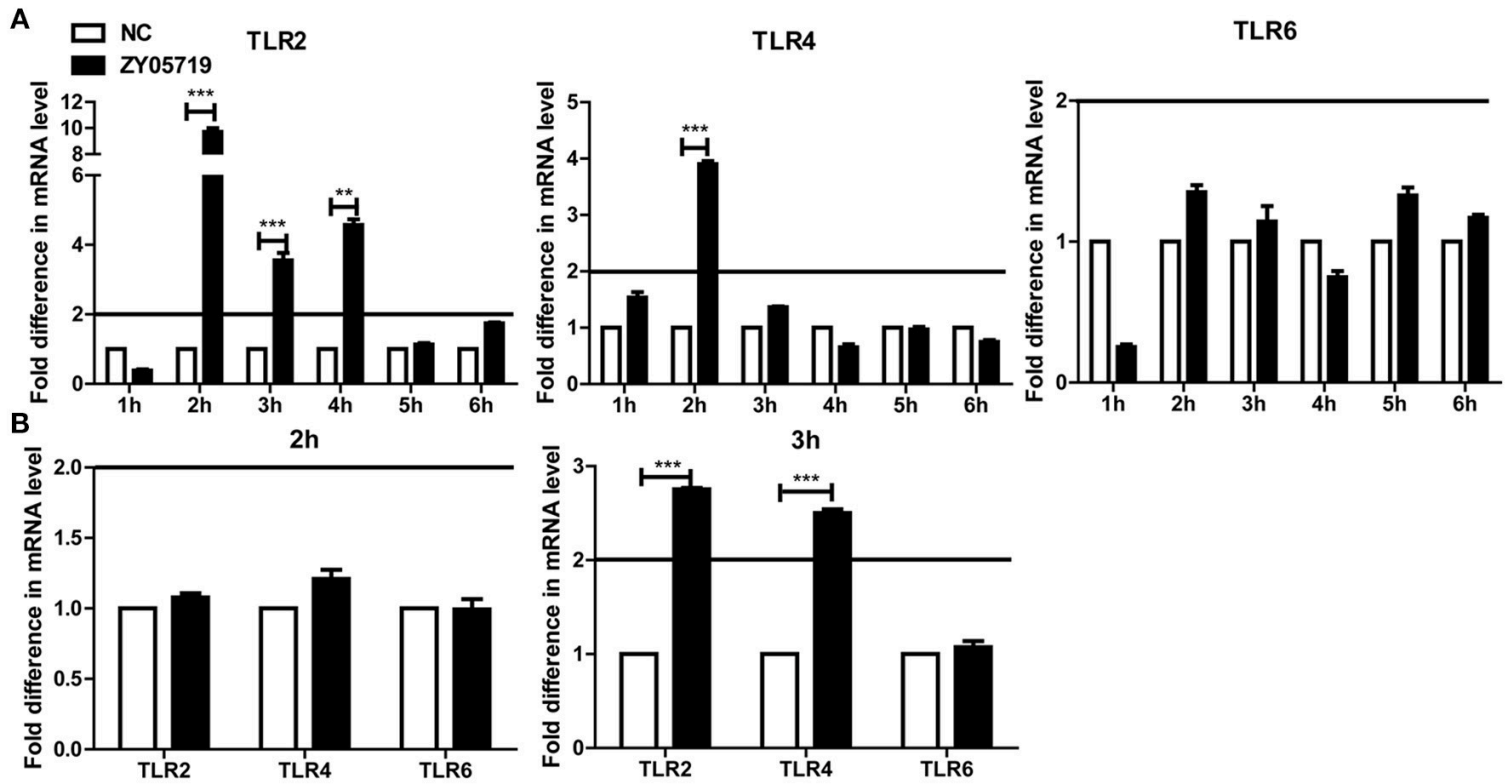
mRNA expression of TLR2, TLR4, and TLR6 *in vivo* and *in vitro*. The GADPH housekeeping gene was used as an internal control, and the relative changes of gene expression were normalized with GADPH using the 2^−ΔΔCt^ method. Mice infected with sterile PBS via intravenous caudal vein injection comprised the negative control (NC), and mRNA expression of NC group was set at 1. **(A)** After injection with SS2 ZY05719 for 1, 2, 3, 4, 5, and 6 h, blood were collected from mice, and RNA was extracted. mRNA expression levels of TLR2, TLR4, and TLR6 were determined by qRT-PCR. **(B)** Neutrophils were infected with SS2 ZY05719 for 2 and 3 h, and RNA was extracted. mRNA expression levels of TLR2, TLR4, and TLR6 were determined by qRT-PCR. The results are depicted as the mean ±SEM (*n* = 3) of three independent experiments; ^**^p < 0.01; ^***^p < 0.001.

Toll-like receptors (TLRs) play an important role in host recognition of pathogens for immune response, and they are thought to participate in NETs induction (39). Transcriptional levels of TLR2, TLR4, and TLR6 were determined to find the relevant cell surface TLRs involved in SS2 recognition. The relative changes of gene expression were normalized with GADPH gene using the 2^−ΔΔCt^ method and mRNA expression levels of health mice blood cells were set as 1. The results showed that TLR2 was significantly up-regulated in blood cells at 2, 3, and 4 h post-infection with SS2 ZY05719. TLR4 mRNA expression at 2 h post-infection with SS2 ZY05719 was 2.8-fold that of blood cells from untreated mice. However, transcription of TLR6 was unchanged post infection with SS2 ZY05719 (Figure 4A). In addition, the transcriptional levels of these three receptors in neutrophils were determined *in vitro*. The mRNA expression levels of untreated neutrophils were set as 1, and mRNA expression levels of TLR2, TLR4, and TLR6 2 h after SS2 ZY05719 stimulation were not up-regulated. Transcriptional levels of neutrophil TLR2 and TLR4 at 3 h post-infection with SS2 ZY05719 were 2.9- and 2.5-fold that of untreated neutrophils, whereas that of TLR6 was unchanged (Figure 4B). These results demonstrated that TLR2 and TLR4 signaling may be involved in the recognition of SS2 leading signal transferred into cells.

### Roles of TLR4 in NETs Formation Induced by SS2

**Figure 5:**
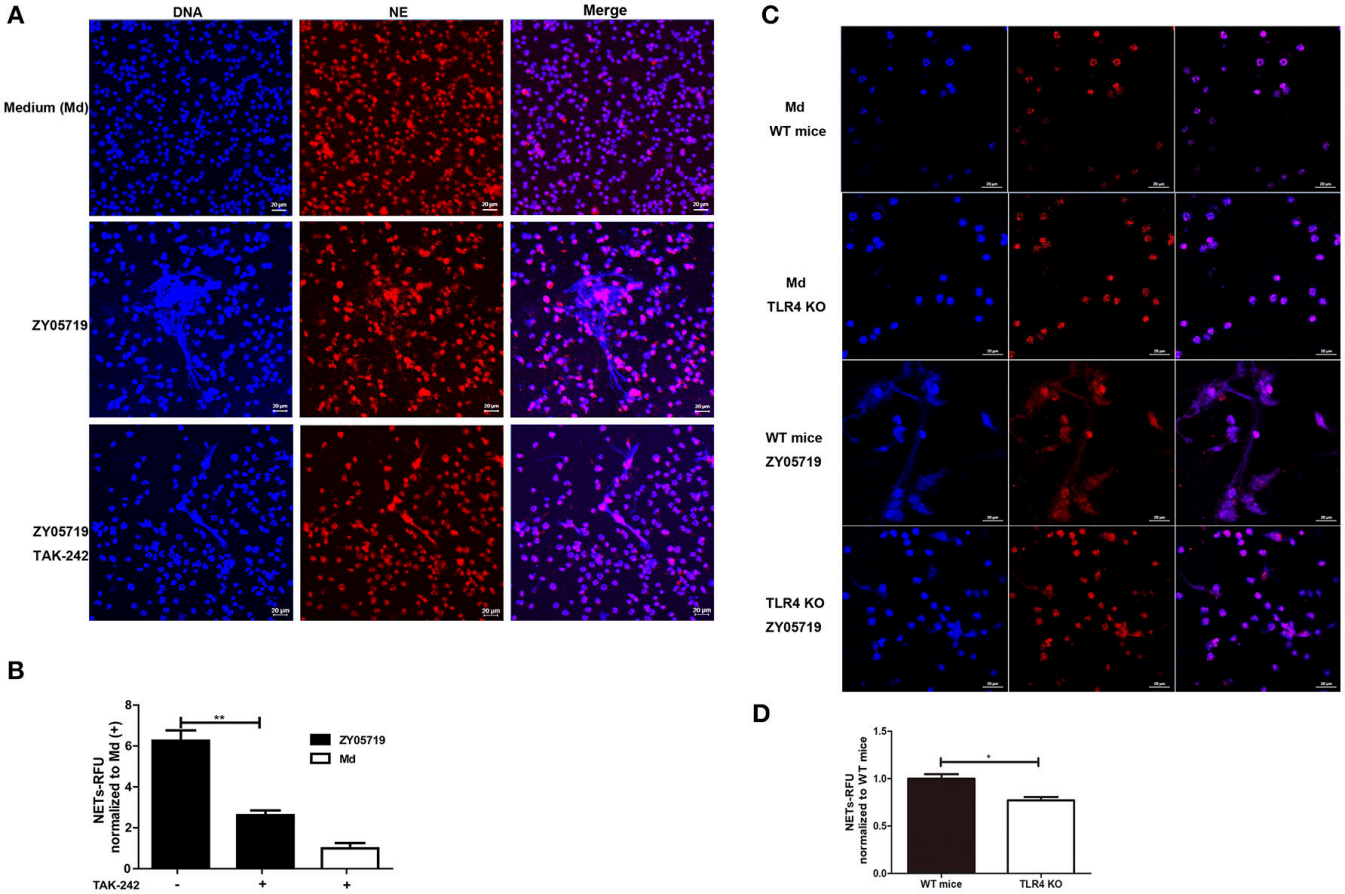
Role of TLR4 signaling in SS2-induced NETs formation. NETs release determination when neutrophils were infected with SS2 ZY05719 and medium (Md) for 3 h. **(A)** Cells were pretreated with or without TLR4 signaling inhibitor TAK-242 and fixed and observed with immunofluorescence using anti-neutrophil elastase (NE) antibody followed by goat anti-rabbit Alexa 568 antibody (red). DNA was stained with DAPI (blue). The results shown are representative of three independent experiments. **(B)** NETs formation was measured as the concentration of extracellular DNA, which was represented as relative fluorescence units (RFU) and normalized to neutrophils pretreated with TAK-242 but without ZY05719 infection. Resulted are depicted as the mean ±SEM (*n* = 3) of three independent experiments; ^**^p < 0.01. **(C)** Neutrophils isolated from wild-type (WT) mice and TLR4 knockout (KO) mice were infected with SS2 ZY05719. Cells were observed with confocal microscope. The results shown are representative of three independent experiments. **(D)** Neutrophils isolated from WT mice and TLR4 KO mice were infected with SS2 ZY05719 and Medium. NETs formation was measured as the concentration of extracellular DNA. ^*^p < 0.05.

TLR2 and TLR4 are generally thought to be the major pattern recognition receptors for Gram-positive bacterial peptidoglycan and Gram-negative bacterial lipopolysaccharide, respectively. Moreover, the interaction between TLR4 and Gram-positive bacterial constituents has been reported in recent years (40). To determine whether TLR4 signaling was involved in SS2 induced NETs formation, neutrophils were pre-incubated with TAK-242 and the cells were infected with SS2 ZY05719. TAK-242 selectively binds with TLR4, and interferes with the interaction between TLR4 and the intracellular adaptor proteins TIRAP/TRAM, thereby inhibiting TLR4 signal transduction and downstream signaling events (41). Neutrophils pretreated with TAK-242 decreased the formation of NETs induced by SS2 infection, which indicated that inhibition of TLR4 signaling negatively affects SS2-induced NETs release to some degree (Figures 5A,B). In addition, NETs formation by neutrophils from TLR4 KO mice decreased by 30% compared with that by neutrophils from WT mice with the stimulation of SS2 ZY05719 (Figures 5C,D). These results proved that SS2 could be recognized by TLR4 and induce NETs formation.

### Roles of ROS and TLR4 Signaling in p38 MAPK and ERK1/2 Activation

**Figure 6:**
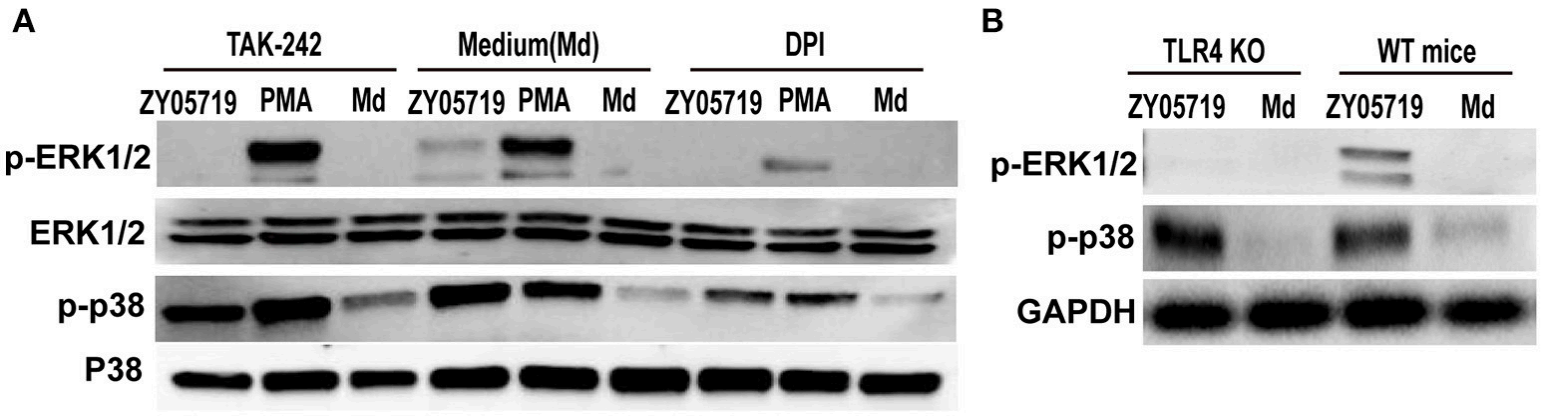
Western blotting analysis of phosphorylation of p38 MAPK and ERK1/2. **(A)** Neutrophils were pretreated with inhibitors of TLR4 signaling (TAK-242) and NADPH oxidase (DPI) for 30 min, and the cells were then incubated with SS2 ZY05719 and PMA for 2 h. Phosphorylation of p38 MAPK (p-p38) and ERK1/2 (p-ERK1/2) were determined with western blotting. **(B)** Neutrophils isolated form wild-type mice and TLR knockout mice were incubated with SS2 ZY05719 and Medium.

Both NADPH oxidase derived ROS and TLR4 signaling are involved in SS2-induced NETs formation. To determine the role of signal transduction in NETs formation, neutrophils were pretreated with inhibition DPI and TAK-242, and phosphorylation events of p38 MAPK and ERK1/2 were assayed by Western blot following SS2 stimulation. When neutrophils were pretreated with DPI to inhibit NADPH oxidase derived ROS, both SS2- and PMA-induced phosphorylation of neutrophil ERK1/2 and p38 MAPK were decreased compared with untreated cells (Figure 6A). This result indicates that NADPH oxidase derived ROS plays an important role in the activation of p38 MAPK and ERK1/2 through both SS2 and PMA stimulation. Importantly, when TLR4 signaling was suppressed, SS2 induced-ERK1/2 activation was inhibited, whereas SS2-induced p38 MAPK activation was not. Moreover, inhibition of TLR4 signaling had no influence on the activation of either p38 MAPK or ERK1/2 induced by PMA (Figure 6A). Neutrophils from TLR4 KO mice were with SS2 and the results showed the activation of ERK1/2 in neutrophils from TLR4 KO mice were suppressed, which confirmed the roles of TLR4 signaling in the activation of ERK1/2 (Figure 6B). These results showed that TLR4 signaling was only necessary for the activation ERK1/2 by SS2 stimulation. Above all, these results demonstrated that SS2 or PMA stimulated neutrophil ROS formation, and the generation of ROS could further activate ERK1/2 and p38 MAPK. TLR4 signaling could activate ERK1/2, which is important for signal transmission during the process of SS2 infection. These results indicate that SS2 could be recognized by TLR4, which is followed by phosphorylation of ERK1/2, and then induction of NETs formation.

## Discussion

SS2 is regarded as an important but neglected emerging zoonotic agent, which leads serious human infection; increasing numbers of human infections have been reported worldwide (42–45). Neutrophil extracellular traps (NETs) are the first innate immunity defense, which play a pivotal role in defense against invasive pathogens (46). The SS2 intermediate pathogenicity A7 and virulent P1/7 strains were reported to stimulate NETs release at 120 min post-infection by fluorescence microscopy (30). In this study, the SS2 strain ZY05719 could not rapidly induce NETs formation (within 30 min), but formation occurred within a duration of 2–4 h, and was dependent on ROS generation from NADPH oxidase. The results showed that SS2 could stimulate NETs release and induce classic suicidal NETosis. However, the exact roles of NADPH oxidase derived ROS on NETs formation induced by SS2 needs further exploration.

Recent studies have reported that NETs composition and the mechanism of NETs formation varies according to the stimulus (47). The vital role of Raf-MEK-ERK signaling pathways in the process of NETs formation induced by PMA was first demonstrated by Hakkim et al. and *Entamoeba histolytica* was reported to induce NETs formation via Raf/MEK/ERK signaling (25, 48). In addition, PMA treatment leads to activation of NADPH oxidase to produce ROS, which then stimulates ERK and p38 MAPK phosphorylation to induce NETs release (27, 49, 50). Gram-negative *Escherichia coli* and *Pseudomonas aeruginosa* were reported to induce ROS-dependent NETs formation via the activation of SAPK/JNK, and this result may account for bacterial LPS (51, 52). The mitogen-activated protein kinase (MAPK) pathway could transfer external and internal signals to regulate cellular events, and ERK1/2, p38 MAPK, JNK/SAPK, and ERK5 are the main constitutes of the MAPK family (26, 53). In this study, SS2 was found to activate p38 MAPK and ERK1/2, but not JNK/SAPK. Inhibition of either the activation of p38 MAPK or ERK1/2 could suppress induction of NETs by SS2 to some degree, demonstrating that both p38 MAPK and ERK1/2 were necessary for SS2-induced NETs formation. However, inhibition of the activation p38 MAPK did not fully suppress SS2 induced NETs formation. The results indicated that activated p38 MAPK may not the key molecule in the process of NETs release induced by SS2 or it might be an additional signal pathway to enhance the capacity of NETs formation induced by SS2. The relationship of p38 MAPK and ERK1/2 in SS2 induced NETs formation needs further study. The related signaling mechanism was likely similar to that of PMA-induced NETs release, but not that of Gram-negative *E. coli*- and LPS-induced NETs formation (27, 54). NETs release induced by Gram-negative bacteria sharing the downstream JNK/SAPK signaling pathway might ascribe to LPS. However, the downstream signaling pathways for NETs induction from other Gram-positive bacteria remain unclear; whether p38 MAPK and ERK1/2 activation are in common for Gram-positive bacteria-induced NETs formation still needs further exploration.

The interaction between ROS generation from NADPH oxidase and SS2-induced p38 MAPK or ERK1/2 activation were detected, and NADPH oxidase inhibition could suppress the activation of p38 MAPK and ERK1/2 to varying degrees. The function of NADPH oxidase derived ROS on NETs formation were consistent with that of ROS produced by PMA and *E. coli*, and ROS may act as a second messenger to transmit signals (54). SS2-and PMA-induced p38 MAPK and ERK1/2 activation and LPS-induced JNK/SAPK activation were downstream of ROS generation, and the subsequent activation of these kinases regulate NETs release (27). The activation of signals involved in NETs formation is complex and diverse due to the different stimuli; this study will add evidences to the molecular mechanism relating to NETs formation.

Neutrophil activation occurs through various membrane receptors, and TLRs are important pattern-recognition receptors (PRRs) for initiation of innate defense through recognition of pathogens (55, 56). In this study, TLR2 and TLR4, were likely involved in NETs formation induced by SS2 according to the results of qRT-PCR, whereas TLR6 was not. It is reported that neutrophils express cell surface receptors TLR2 and TLR4, and TLR ligation modulates varieties of neutrophil responses including NETs release (57). In addition, NETs formation is associated with high TLR2 and TLR4 expression levels (58). TLR2 was reported to recognize components of Gram-positive bacteria such as peptidoglycan and lipoteichoic acid (LTA) (59). TLR2 was reported to regulate NETs release during Gram-positive skin infection (39). TLR4 mediates host responses to Gram-negative bacterial lipopolysaccharide (60). In addition, TLR4 is required for the recognition of LTA of Gram-positive bacteria such as *S. aureus* (61). TLR2 and TLR4 expression are reported to be involved in the virulence of *Streptococcus pyogenes* (60). TLR4 signaling is the response signal for LPS-induced NETs formation (54). F protein of respiratory syncytial virus can bind with TLR4 and lead to NETs production (62). SS2 infection would up-regulate the transcription of TLR2 and TLR4, but not TLR6, *in vitro* and *in vivo*. TLR2 is an important receptor in NETs formation and SS2 infections (63, 64). The expression of TLR2 was higher and more lasting in blood after SS2 infection, indicating TLR2 might play a significant role in NETs induction by SS2. The function of TLR2 needs to be further studied. In addition, involvement of TLR4 in NETs formation was previously reported, and blocking TLR4 signaling could suppress the activation of JNK/SAPK in the process of LPS induced NETs formation (54). Therefore, the roles of TLR4 signaling in NETs formation were studied. In this study, SS2 induced TLR4 signaling was demonstrated, and blocking TLR4 signaling could suppress SS2-induced ERK1/2 activation and NETs release, but p38 MAPK activation was unsuppressed. However, inhibition of TLR4 signaling had no influence on that induced by PMA, indicating that PMA-induced activation of p38 MAPK and ERK1/2 are not initiated with TLR4 signaling. To further confirm the role of TLR4 signaling in SS2 induced NETs formation, neutrophils isolated from TLR4 KO mice were infected with SS2 ZY05719 and the results showed that neutrophils from TLR4 KO mice were less likely to release NETs and activate ERK1/2 with the stimulation of SS2 ZY05719. Moreover, SS2 could be recognized by TLR4, which subsequently activated ERK1/2 and result in NETs release. TLR2 is involved in the recognition of SS2, and its function in NETs induction by SS2 needs further study.

The battle between SS2 and NETs is a process of checks and balances, and the winning side will determine disease development. In this study, we found that SS2 could be recognized by TLR2 and TLR4. TLR4 signaling was studied and found to be associated with phosphorylation of neutrophil ERK1/2 and SS2-induced NETs formation. In addition, SS2-induced NETs release was dependent on ROS from NADPH oxidase. SS2 could stimulate neutrophil ROS generation; this was followed by p38 MAPK and ERK1/2 activation, which was necessary for SS2-induced NETs release. This study could contribute to understanding the downstream signaling SS2 induction of NETs, which will aid further studies of SS2 pathogenicity.

## Author Contributions

All authors have significant contributions to the completion of the manuscript. FM and HF: conception and design of the work; FM, XC, and GW: acquisition and analysis the data; FM, GW, and HZ: interpretation of data for the work; FM, ZM, HL, and HF: drafting and revising the work; FM, XC, GW, HZ, ZM, HL, HF: final approval and agreement to be accountable for all aspects of the work.

### Conflict of Interest Statement

The authors declare that the research was conducted in the absence of any commercial or financial relationships that could be construed as a potential conflict of interest.
